# Epidemiological characteristics and antimicrobial resistance of pathogens isolated from blood cultures in southern Jiangxi, China, 2020–2024

**DOI:** 10.3389/fcimb.2025.1727877

**Published:** 2026-01-13

**Authors:** Zhengting Liu, Liqin Zhang, Jian Zou

**Affiliations:** Department of Clinical Laboratory, Ganzhou People’s Hospital, Ganzhou, China

**Keywords:** antimicrobial agents, blood culture, drug resistance, multidrug-resistant bacteria, pathogen spectrum

## Abstract

**Objective:**

This study aimed to analyze the distribution characteristics and dynamic trends of antimicrobial resistance among pathogens isolated from blood cultures of adult patients at a large tertiary hospital in southern Jiangxi Province, China, from 2020 to 2024, in order to provide evidence-based guidance for the prevention and treatment of bloodstream infections.

**Methods:**

This study conducted a retrospective analysis of non-repetitive isolates from blood cultures of adult patients at a large tertiary hospital in southern Jiangxi Province between 2020 and 2024. Statistical analysis was performed using WHONET 5.6 and SPSS Statistics 30 software.

**Results:**

This study included a total of 3,695 pathogenic bacteria, with Gram-negative bacteria predominating (61.92%,2,288/3,695). Among Gram-negative bacteria, *Escherichia coli* (28.99%,1,071/3,695) and *Klebsiella pneumoniae* (14.72%,544/3,695) were the most prevalent. Antimicrobial resistance analysis revealed that the detection rate of carbapenem-resistant *Klebsiella pneumoniae (CRKP)* surged sharply from 1.2% (1/82) in 2020 to 21.8% (26/119) in 2024; the detection rate of carbapenem-resistant *Acinetobacter baumannii (CRAB)* increased from 33.3%(5/15) in 2020 to 76.5% (13/17) in 2024. In contrast, the detection rate of methicillin-resistant *Staphylococcus aureus (MRSA)* decreased significantly from 24.3% (17/73) in 2020 to 13.5% (10/74) in 2024. Among *Enterococci*, the rate of high-level gentamicin resistance (HLGR, defined as resistance to 500 µg/ml gentamicin) in *Enterococcus faecium* increased significantly, from 10% (1/10) in 2020 to 66.7% (14/21) in 2024, and vancomycin-resistant *Enterococcus faecium* (VREfm) was detected at a rate of 9.5% (2/21) in 2024.

**Conclusion:**

In a tertiary hospital in southern Jiangxi, China, Gram-negative bacteria predominate among Patients with positive blood cultures, with sharply rising detection rates of *CRKP*, *CRAB*, and *VREfm* posing a public health threat. Meanwhile, the declining prevalence of *MRSA* indicates that infection control measures are effective against Gram-positive bacteria. Therefore, continuous surveillance of drug-resistant bacteria is essential, and antimicrobial stewardship measures must be implemented immediately to curb their spread. A limitation of this study is that it was conducted at a single center, which may restrict the generalizability of the findings to other regions.

## Introduction

Bloodstream infections (BSIs) are life-threatening conditions associated with high mortality and substantial healthcare burden (Jeon et al., 2012; Goto and Al-Hasan, 2013; Massart et al., 2021). The selection of effective empirical antimicrobial therapy relies heavily on local epidemiological data, as significant geographic variations exist in the predominant pathogens and their resistance profiles. For instance, while Gram-positive bacteria, particularly *coagulase-negative staphylococci*, are reported as predominant in some regions like Italy (Licata et al., 2020), Gram-negative bacteria, such as *Escherichia coli*, are more common in others, including Korea (Choi et al., 2022). In China, similar regional disparities are evident, with studies from the northwest and southwest reporting different leading pathogens (Liu et al., 2025; Long et al., 2025).

This pronounced geographical heterogeneity indicates that relying on localized epidemiological surveillance data is crucial for guiding empirical clinical treatment. However, there is currently a significant lack of comprehensive and up-to-date research data on the distribution of pathogens and the evolving trends of antimicrobial resistance in bloodstream infections specifically in Jiangxi Province, southern China, which constitutes a critical knowledge gap. Concurrently, the global threat of bacterial antimicrobial resistance is becoming increasingly severe. Studies indicate that in 2019, antimicrobial resistance directly caused 1.27 million deaths and was associated with a total of 4.95 million deaths worldwide (Murray et al., 2022). The World Health Organization (WHO) further warns that without effective intervention, resistance could lead to over 10 million deaths annually by 2050 (World Health Organization, 2021). Therefore, continuous monitoring of regional pathogen profiles and dynamic resistance patterns is fundamental for developing precise infection control strategies and optimizing antimicrobial therapy.

Against this backdrop, this study aimed to systematically analyze the pathogen spectrum and dynamic antimicrobial resistance trends among isolates from blood cultures at a large tertiary hospital in southern Jiangxi Province from 2020 to 2024. By elucidating the local epidemiological characteristics and revealing the prevalence and temporal changes of key multidrug-resistant bacteria, this research seeks to provide critical data support and evidence-based guidance for optimizing local empirical antimicrobial treatment regimens, strengthening antimicrobial stewardship, and enhancing infection control measures.

## Methods

### Study design

This retrospective laboratory surveillance study analyzed all bacterial isolates detected from blood cultures of adult patients (≥18 years old) at Ganzhou People’s Hospital between January 1, 2020, and December 31, 2024. The objective was to investigate the distribution of clinically significant pathogens in blood cultures and their evolving antimicrobial resistance patterns, thereby reflecting the microbiological characteristics of suspected bloodstream infections in this region.

### Bacterial strain selection

The screening criteria for blood culture isolates in this study are as follows: Inclusion criteria are isolates identified as pathogenic bacteria (e.g., *Staphylococcus aureus*, *Escherichia coli*, *Klebsiella pneumoniae*, etc.) from blood cultures of adult patients aged ≥18 years, specifically the first non-replicated isolate from each patient meeting these conditions.

Exclusion criteria included common skin colonizers typically considered contaminants (e.g., *Propionibacterium acnes*, *Bacillus* spp., *Micrococcus* spp., etc.); *Coagulase-negative Staphylococci (CoNS)* were included only if clinically significant contamination was confirmed by the attending physician based on clinical context.

### First non-replicated isolate

In this study, the “first non-replicated isolate” refers to “the first isolate per patient per microbial species,” operationally defined as: the earliest chronologically obtained isolate of each specific microbial species (including bacteria and fungi) recovered from an individual patient during the entire study period (2020–2024).

### Microbiology methods

Blood samples were inoculated into BACTEC™ Plus Aerobic/F and Lytic/10 Anaerobic/F blood culture bottles (Becton, Dickinson, USA) and incubated in the BD BACTEC™ system at 35 ± 1 °C for up to 5 days or until flagged positive. Positive bottles underwent Gram staining and were subcultured onto solid media: blood agar plates, chocolate agar plates, and China Blue agar (Detgerm, China). Subcultures on China Blue agar were incubated at 35 ± 2 °C in ambient air for 18–24 hours. Blood agar and chocolate agar plates were incubated at 35 ± 2 °C in a 5% CO_2_ atmosphere for 18–24 hours to support the growth of fastidious organisms.

Bacterial identification was performed using the VITEK 2-Compact system or the VITEK MS Matrix-Assisted Laser Desorption/Ionization Time-of-Flight Mass Spectrometry system (BioMérieux, France). Antimicrobial susceptibility testing was primarily performed using the VITEK^®^ 2 Compact fully automated system (with dedicated AST cards; bioMérieux, France) for minimum inhibitory concentration determination. In addition, the Kirby–Bauer disk diffusion method was used as a supplementary and verification approach: bacterial suspensions were adjusted to a 0.5 McFarland standard and evenly spread onto Mueller–Hinton agar plates (Detgerm,China). After applying antimicrobial discs, the plates were incubated at 35 ± 2 °C in ambient air for 16–24 hours. For fastidious bacteria, corresponding culture media were used for AST: for example, *Haemophilus influenzae* was tested on HTM agar plates and incubated in a 5% CO_2_ atmosphere. All AST cards and antibiotic discs were stored and used strictly in accordance with the manufacturers’ instructions and within their expiration dates. Susceptibility results were interpreted in strict compliance with the breakpoints specified in the 34th edition of the Clinical and Laboratory Standards Institute (CLSI) guidelines.

### Quality control

To ensure data accuracy and reliability, this study routinely employed the following American Type Culture Collection (ATCC) standard strains for quality control: *Staphylococcus aureus* ATCC 25923 and ATCC 29213, *Escherichia coli* ATCC 25922, *Pseudomonas aeruginosa* ATCC 27853, *Streptococcus pneumoniae* ATCC 49619, and *Haemophilus influenzae* ATCC 49247. All quality control tests were conducted concurrently with clinical isolates at a frequency of once per week, with results falling within the acceptable ranges specified by CLSI guidelines. All operations during the study period strictly adhered to laboratory standard operating procedures. Internal audit records confirmed no deviations, and all strain data included in the analysis passed corresponding quality control.

### Statistical analysis

Bacterial and fungal identification and antimicrobial susceptibility data were uniformly managed, analyzed, and screened for duplicate isolates using WHONET 5.6 software. The specific workflow is as follows: (1) Data Import: Standardized data files containing patient demographic information, microbial identification results, and antimicrobial susceptibility testing results were imported into the software. (2) Analysis Configuration: The “First Isolate per Patient” analysis mode was enabled. This function automatically filters the dataset, retaining only the earliest isolate per microbial species per patient for subsequent statistical analysis. Meanwhile, relevant analysis options were configured according to the study objectives, such as filtering by specific pathogen categories and antimicrobial classes. (3) Data Analysis and Export: Preliminary epidemiological and antimicrobial resistance analyses were performed using the software. Statistical reports were generated, and the processed dataset was exported for further statistical testing.

Categorical variables are presented as frequencies (n) and percentages (%). Comparisons of categorical data between groups were performed using the Pearson chi-square test or Fisher-Freeman-Halton exact test. All tests employed a two-tailed P-value < 0.05 as the criterion for statistical significance.

All statistical analyses were performed using SPSS Statistics version 30.0 (IBM Corporation, USA). Graphs and charts were generated using OriginPro 2023 software (Electronic Arts, USA).

## Results

### Baseline characteristics and pathogen spectrum

Between 2020 and 2024, a total of 3,695 first, non-repetitive isolates from blood cultures were collected from adult patients (≥18 years). The baseline characteristics of the patients are presented in **Table 1**. Most isolates were from male patients (58.9%) and individuals aged 45 years or older (86.5%), with nearly half (44.7%) from patients aged ≥65 years. The majority of isolates (77.7%) were obtained from general wards, with the remaining 22.3% originating from intensive care units (ICUs) at the time of blood culture sampling.

**Table 1:** Baseline characteristics of patients with positive blood cultures.

Variables	Number of strains (n)	Percentage (%)
Gender		
Male	2177	58.92
Feamale	1518	41,08
Age (years)		
18-44	499	13.5
45-64	1544	41.79
≥65	1652	44.71
Ward		
ICU	824	22.3
General ward	2871	77.7
Total	3695	100

Gram-negative bacteria were the predominant group (61.9%), followed by Gram-positive bacteria (35.0%) and fungi (3.1%). *Escherichia coli* (29.0%) and *Klebsiella pneumoniae* (14.7%) were the most frequently isolated pathogens. Among Gram-positive bacteria, *coagulase-negative staphylococci* (14.5%) and *Staphylococcus aureus* (9.7%) were most common (**Table 2**).

**Table 2:** Distribution of pathogenic organisms isolated from patients with positive blood cultures.

Variables	Number of strains (n)	Percentage (%)
Gram negatives	2288	61.92
*Escherichia coli*	1071	28.99
*Klebsiella pneumoniae*	544	14.72
*Pseudomonas aeruginosa*	101	2.73
*Enterobacter cloacae*	99	2.68
*Acinetobacter baumannii*	87	2.35
Others	386	10.45
Gram positives	1293	34.99
*coagulase-negative staphylococci*	535	14.48
*Staphylococcus aureus*	358	9.69
*Enterococcus faecium*	88	2.38
*Enterococcus faecalis*	61	1.65
Others	251	6.79
Fungers	114	3.09
*Candida albicans*	38	1.03
*Candida parapsilosis*	30	0.81
*Candida tropicalis*	27	0.73
Others	19	0.52
Total	3695	100

Subgroup analysis (**Supplementary Figure 1S**) revealed distinct epidemiological patterns. *Escherichia coli* was the leading pathogen across all age groups, with its relative proportion increasing with patient age. Pathogen distribution differed markedly between clinical settings: ICU isolates showed greater diversity, with higher proportions of *Acinetobacter baumannii*, *Enterococcus faecium*, and *Burkholderia cepacia* compared to general wards.

**Figure 1:**
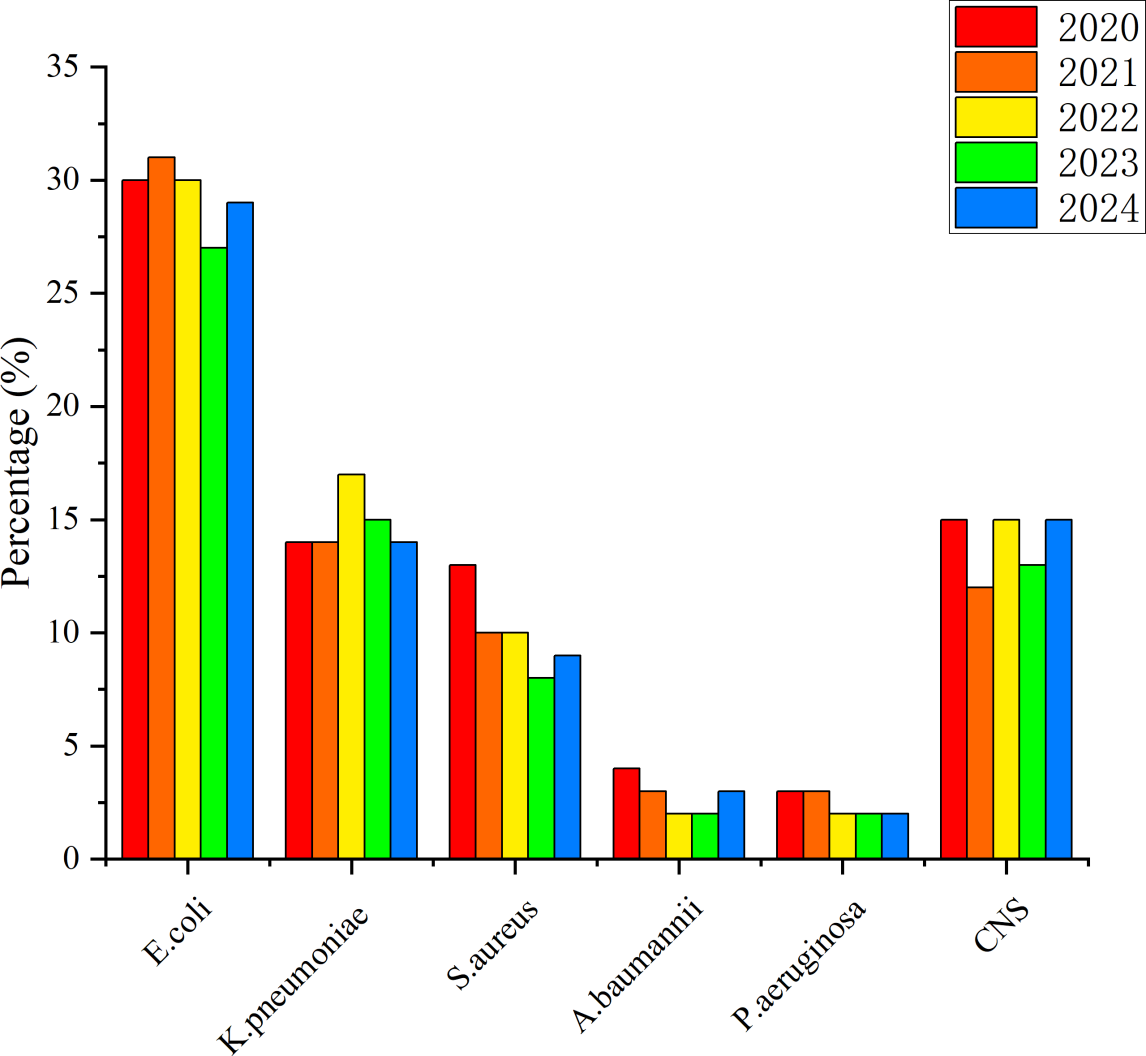
Distribution characteristics of pathogens isolated from blood cultures, 2020–2024.

The annual distribution of the six most prevalent pathogens is shown in **Figure 1**. The proportions of *Klebsiella pneumoniae* and *CNS* remained relatively stable over the five-year period. In contrast, the proportions of *Escherichia coli*, *Staphylococcus aureus*, *Pseudomonas aeruginosa*, and *Acinetobacter baumannii* exhibited a gradual declining trend.

### Changes in antimicrobial resistance

#### Predominant gram-negative bacteria

Detailed annual resistance rates for *Escherichia coli* and *Klebsiella pneumoniae* are provided in **Supplementary Table 1S**. *Escherichia coli* resistance to most antimicrobials remained stable, with no significant difference in ESBL-production rates across the study years (*p* = 0.052). Resistance to carbapenems and amikacin was consistently low (<4%), and all isolates were susceptible to tigecycline.

In contrast, *Klebsiella pneumoniae* exhibited alarming increases in resistance. Carbapenem resistance (ertapenem and imipenem) surged significantly, from approximately 1.2% in 2020 to over 20% in 2024 (*p* < 0.001). Significant upward increases were also observed for multiple other drug classes, including cephalosporins, piperacillin-tazobactam, amikacin, and levofloxacin (all *p* < 0.05). All *Klebsiella pneumoniae* isolates remained susceptible to tigecycline.

For non-fermentative bacilli (**Supplementary Table 2S**), the resistance rates of *Pseudomonas aeruginosa* to most tested agents showed no statistically significant differences across the study years (*p* > 0.05). Conversely, *Acinetobacter baumannii* resistance escalated dramatically across nearly all antimicrobial categories. Resistance rates to key agents such as cefoperazone-sulbactam, carbapenems (imipenem/meropenem), and fluoroquinolones increased significantly, with most exceeding 75% by 2024 (*p* < 0.05). All *Acinetobacter baumannii* isolates remained susceptible to tigecycline.

#### Predominant gram-positive bacteria

Detailed resistance data for *Staphylococcus* spp. and *Enterococcus* spp. are shown in **Supplementary Tables 3S**, **4S** respectively. *Staphylococcus aureus* maintained very high resistance to penicillin (>90%). The prevalence of MRSA decreased to 13.5% in 2024, though the difference across the five years was not statistically significant (*p* = 0.303). All isolates were susceptible to linezolid, vancomycin, and tigecycline.

*CNS* displayed higher overall resistance. Methicillin resistance (MRCNS) remained persistently high (74.7-85.1%) without a significant difference across the years. A notable exception was a significant decline in resistance to trimethoprim-sulfamethoxazole (*p* < 0.001).

*Enterococcus faecium* showed concerning changes, with significant increases in resistance to penicillin (p = 0.030) and high-level gentamicin (rising from 10.0% to 66.7%, p = 0.011). *VREfm* emerged in 2022 and was detected at 9.5% in 2024. *Enterococcus faecalis* exhibited a different profile, with significant increases in high-level gentamicin and levofloxacin resistance, but maintained full susceptibility to vancomycin.

### Consolidated changes in key resistance phenotypes

**Figure 2:**
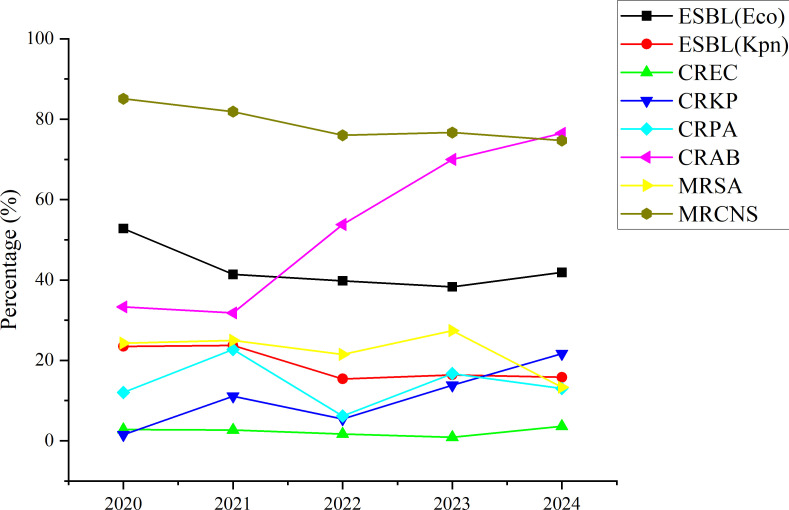
Changes in prevalence of key antimicrobial resistance phenotypes among blood culture isolates, 2020–2024.

The temporal trends of major antimicrobial resistance phenotypes are consolidated in **Figure 2**. The most prominent finding was the rapid and concurrent increase in *CRKP* and *CRAB*. In contrast, *MRSA*, *MRCNS*, and *ESBL*-producing *Escherichia coli* and *Klebsiella pneumoniae* showed decreasing trends, while the prevalence of carbapenem-resistant *Escherichia coli* (*CREC*) remained at a relatively low level throughout the study period.

## Discussion

This five-year retrospective analysis at a large tertiary hospital in southern Jiangxi reaffirms the predominance of Gram-negative bacteria among pathogens isolated from blood cultures locally, consistent with major Chinese surveillance reports (e.g., CHINET data showing 67.5% Gram-negative predominance nationwide) (Hu et al., 2022). However, notable geographic and demographic variations exist. Pediatric data from the same province reported Gram-positive predominance (70.7%) (Zhou et al., 2023), while Italian surveillance also found Gram-positive bacteria to be most frequent (57.8%) (Licata et al., 2020). These contrasts underscore how pathogen distribution is shaped by regional epidemiology, age structure, and clinical settings.

Subgroup variations further illustrate this point. The significantly higher proportion of *Escherichia coli* infections among female patients and its increasing burden with age, along with the higher prevalence of *Enterococcus* spp. and *Acinetobacter baumannii* in ICU patients, likely reflect differences in underlying conditions, immune status, and exposure to invasive procedures (Zeng et al., 2024).

Antimicrobial resistance patterns revealed critical challenges. While *Escherichia coli* resistance to several first-line agents (e.g., cefuroxime, ceftriaxone, levofloxacin, co-trimoxazole) remains high – supporting earlier suggestions that these drugs be avoided in empirical therapy for suspected *Escherichia coli* BSI (Liu et al., 2025)– carbapenem resistance stayed relatively low (<3.6%) without a significant upward trend. This suggests carbapenems retain utility for serious *Escherichia coli* infections locally, though the rate still slightly exceeds the national average (Hu et al., 2022; Qin et al., 2024), possibly indicating regional prescription or transmission pressures.

In stark contrast, *Klebsiella pneumoniae* exhibited a rapid and worrisome expansion of resistance. Dramatic increases in resistance to piperacillin/tazobactam, cefoperazone/sulbactam, amikacin, levofloxacin, and particularly to carbapenems (with *CRKP* rising from 1.2% to 21.8% over the study period) have severely narrowed therapeutic options. The swift dissemination of *CRKP* is likely driven by both carbapenem selection pressure and the hospital persistence of high-risk clones such as ST11, which has been linked to outbreaks across Asia (Jin et al., 2021). This leaves only a few last-line agents (e.g., tigecycline, polymyxins, newer β-lactamase inhibitor combinations) as viable options (Durante-Mangoni et al., 2019).

Among non-fermenters, *Pseudomonas aeruginosa* generally remained susceptible to common anti-pseudomonal drugs, whereas *Acinetobacter baumannii* displayed extensive multidrug resistance. The steep rise in *CRAB* (from 33.3% to 76.5%) highlights its formidable capacity for nosocomial persistence and spread, attributable to environmental resilience, efflux pump overexpression, and the production of OXA-type carbapenemases (Oh et al., 2020; Liu et al., 2022). Given the current extremely limited therapeutic options, all *Acinetobacter baumannii* strains in this study maintained 100% *in vitro* susceptibility to tigecycline, suggesting these agents may become critical drugs for addressing such multidrug-resistant infections (Pormohammad et al., 2020).

Notably, the declining MRSA detection rate (24.3% to 13.5%) contrasts sharply with the escalating Gram-negative resistance crisis and mirrors national and international surveillance trends (Kuehn, 2019; Qin et al., 2024). This improvement likely reflects the cumulative impact of antimicrobial stewardship and strengthened infection-control measures in our hospital (Wiese-Posselt et al., 2023; Tong et al., 2025). Conversely, *coagulase-negative staphylococci* maintained high resistance levels, underscoring the ongoing difficulty in distinguishing contamination from true infection and in preventing device-related bloodstream events.

*Enterococci* displayed species-specific resistance patterns. *Enterococcus faecium* showed markedly elevated resistance to high-level gentamicin and penicillins, challenging the utility of classic ampicillin-gentamicin synergy for many infections. Although *VREfm* emerged and reached 9.5% by 2024 – higher than some Chinese reports (Long et al., 2025; Wang et al., 2025)but lower than rates in the United States (Mendes et al., 2016). This reflects substantial geographical variation in *VREfm* prevalence, warranting continued close monitoring in this region.

However, this study also has several limitations. First, as a single-center study, the generalizability of its findings may be limited; nevertheless, it provides a precise epidemiological baseline for local and similar healthcare settings to inform prevention and control measures. Second, the case definition employed in this study was laboratory-based. Although this definition aimed to identify clinically relevant pathogens, it may not fully align with the comprehensive clinical diagnostic criteria for bloodstream infection. Therefore, the results of this study primarily reflect the epidemiological characteristics of microbiologically confirmed pathogens from blood cultures. Finally, this study did not include molecular investigations, and thus could not confirm resistance mechanisms or clonal transmission relationships at the genetic level—this represents an important direction for future research.

## Conclusion

The findings of this study indicate that bloodstream infections among adults in southern Jiangxi Province, China, are increasingly dominated by Gram-negative bacteria, with alarming rates of CRKP, CRAB, and VREfm emerging as a significant public health threat. Although the declining prevalence of MRSA reflects the effectiveness of infection control measures against Gram-positive bacteria, the current focus of prevention and control efforts must urgently shift toward multidrug-resistant Gram-negative bacteria. We emphasize the imperative to immediately strengthen continuous surveillance of resistant bacteria, strictly enforce antimicrobial stewardship, and implement targeted infection control strategies to effectively curb their spread.

## Data Availability

The original contributions presented in the study are included in the article/**Supplementary Material**. Further inquiries can be directed to the corresponding author.
