# Supplementary material for: Epidemiological characteristics and antimicrobial resistance of pathogens isolated from blood cultures in southern Jiangxi, China, 2020–2024
Source: Front Cell Infect Microbiol. 2026 Jan 13;15:1727877. doi: 10.3389/fcimb.2025.1727877 (PMC12835207; doi:10.3389/fcimb.2025.1727877)
Supplement: Supplementary file 1 [file Table1.docx]

**SupplementaryTables**
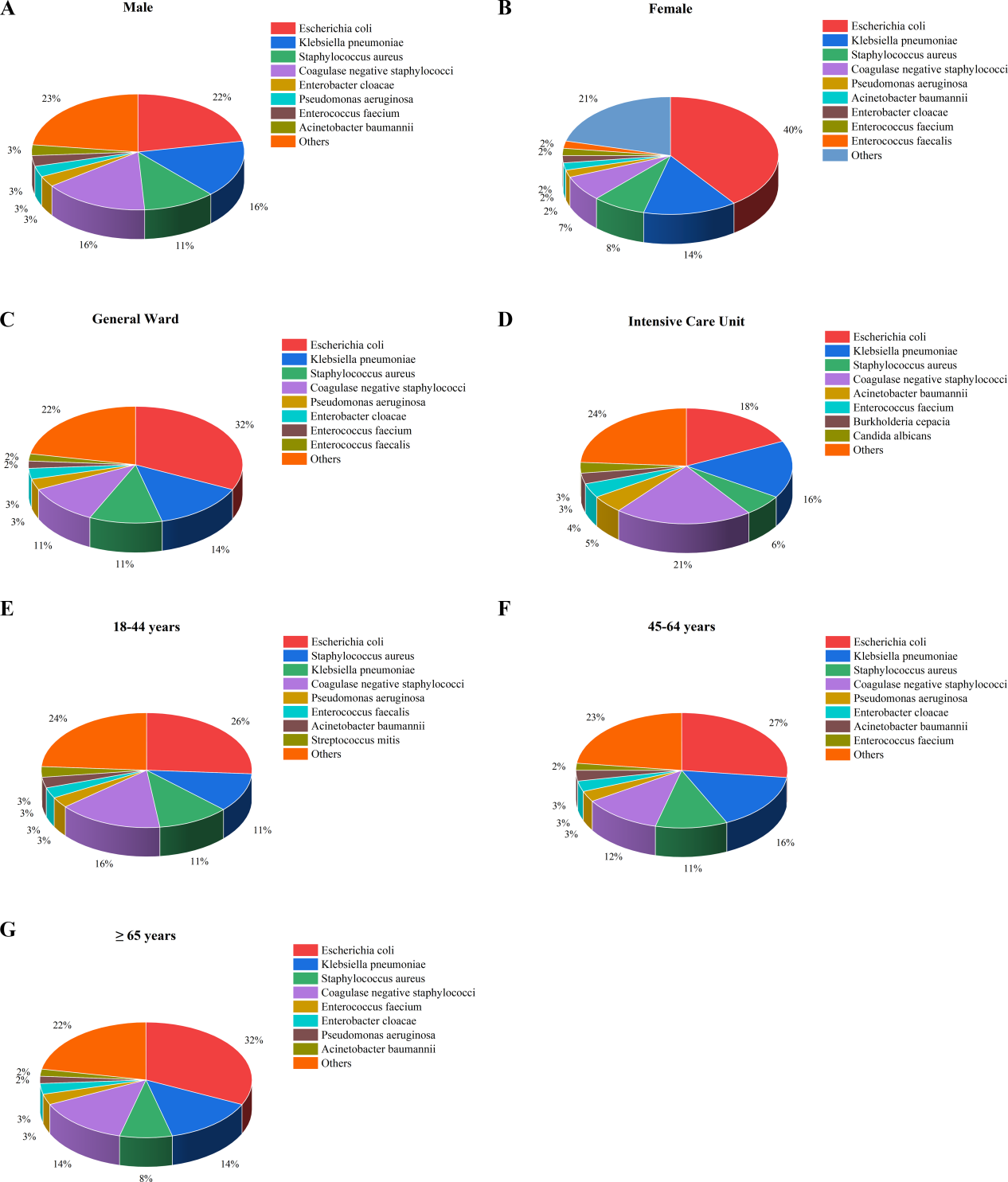


Figure 1S Distribution Characteristics of Pathogens Isolated from Blood Cultures Among Different Populations

Table 1S Antimicrobial Resistance Rates of *Escherichia coli* and *Klebsiella pneumoniae* Isolates, 2020–2024

| ****Antibiotics**** | **Escherichia coli** | | | | | | | | | | **95%CI** | ***p*** |
| --- | --- | --- | --- | --- | --- | --- | --- | --- | --- | --- | --- | --- |
|  | 2020(n = 173) | | 2021(n = 218) | | 2022(n = 225) | | 2023(n = 213) | | 2024 (n = 242) | |  |  |
|  | Resistant | Non-resistant | Resistant | Non-resistant | Resistant | Non-resistant | Resistant | Non-resistant | Resistant | Non-resistant |  |  |
| ESBL | 52.8 | 47.2 | 41.4 | 58.6 | 39.8 | 60.2 | 38.3 | 61.7 | 41.9 | 58.1 | 0.049-0.057 | 0.052 |
| AMC | 10.4 | 89.6 | 11.7 | 88.3 | 10.4 | 89.6 | 12.1 | 87.9 | 13.3 | 86.7 | 0.841-0.855 | 0.850 |
| CSL | 5.6 | 94.4 | 4.9 | 95.1 | 4.8 | 95.2 | 2.3 | 97.7 | 5.6 | 94.4 | 0.386-0.406 | 0.396 |
| TZP | 5 | 95 | 4.9 | 95.1 | 5.2 | 94.8 | 4.1 | 95.9 | 9.7 | 90.3 | 0.119-0.132 | 0.125 |
| CXM | 48.3 | 51.7 | 46.4 | 53.6 | 41.6 | 58.4 | 41.3 | 58.7 | 46.8 | 53.2 | 0.497-0.516 | 0.509 |
| CAZ | 21.8 | 78.2 | 18.6 | 81.4 | 15.6 | 84.4 | 13 | 87 | 19.8 | 80.2 | 0.138-0.152 | 0.145 |
| CRO | 45.8 | 54.2 | 46 | 54 | 42 | 58 | 40.4 | 59.6 | 47.2 | 52.8 | 0.585-0.604 | 0.595 |
| FEP | 19.6 | 80.4 | 7.1 | 92.9 | 9.1 | 90.9 | 7.3 | 92.7 | 12.9 | 87.1 |  | <0.001 |
| FOX | 13.3 | 86.7 | 9.4 | 90.6 | 6.9 | 93.1 | 7.9 | 92.1 | 12.5 | 87.5 | 0.144-0.159 | 0.152 |
| ETP | 2.7 | 97.3 | 2.7 | 97.3 | 1.7 | 98.3 | 0.9 | 99.1 | 3.6 | 96.4 | 0.329-0.347 | 0.338 |
| IPM | 2.8 | 97.2 | 2.7 | 97.3 | 1.3 | 98.7 | 0.9 | 99.1 | 3.6 | 96.4 | 0.234-0.251 | 0.243 |
| AMK | 2.8 | 97.2 | 1.8 | 98.2 | 0.9 | 99.1 | 1.8 | 98.5 | 1.2 | 98.8 | 0.599-0.618 | 0.608 |
| LEV | 38.2 | 61.8 | 37.9 | 62.1 | 37.7 | 62.3 | 40.6 | 59.4 | 41.1 | 58.9 | 0.902-0.913 | 0.905 |
| SXT | 46.6 | 53.4 | 50 | 50 | 42.9 | 57.1 | 47 | 53 | 48.4 | 51.6 | 0.599-0.618 | 0.610 |
| TGC | 0 | 100 | 0 | 100 | 0 | 100 | 0 | 100 | 0 | 100 |  |  |
| **Antibiotics** | Klebsiella pneumoniae | | | | | | | | | | **95%CI** | ***p*** |
|  | 2020 ( n = 82) | | 2021 ( n = 99) | | 2022 ( n = 128) | | 2023 (n = 116) | | 2024 ( n = 119) | |  |  |
|  | Resistant | Non-resistant | Resistant | Non-resistant | Resistant | Non-resistant | Resistant | Non-resistant | Resistant | Non-resistant |  |  |
| ESBL | 23.5 | 76.5 | 23.7 | 76.3 | 15.4 | 84.6 | 16.4 | 83.6 | 15.8 | 84.2 | 0.376-0.395 | 0.386 |
| AMC | 11 | 89 | 19 | 81 | 12.3 | 87.7 | 21.6 | 78.4 | 23.3 | 76.7 | 0.058-0.068 | 0.062 |
| CSL | 7.2 | 92.8 | 14 | 86 | 6.9 | 93.4 | 16.1 | 83.9 | 24 | 76 |  | <0.001 |
| TZP | 13.3 | 86.7 | 16 | 84 | 9.2 | 90.8 | 20.3 | 79.7 | 25.6 | 74.4 | 0.010-0.014 | 0.012 |
| CXM | 22.9 | 77.1 | 31 | 69 | 23.1 | 76.9 | 31.4 | 68.6 | 35.5 | 64.5 |  | <0.001 |
| CAZ | 17.1 | 82.9 | 29 | 71 | 15.4 | 84.6 | 23.9 | 76.4 | 27.3 | 72.7 | 0.062-0.072 | 0.067 |
| CRO | 25.8 | 74.2 | 32 | 68 | 20.8 | 79.2 | 29.1 | 70.9 | 34.7 | 65.3 | 0.151-0.166 | 0.159 |
| FEP | 16.9 | 83.1 | 27 | 73 | 13.8 | 86.2 | 24.6 | 75.4 | 31.4 | 68.6 | 0.008-0.011 | 0.010 |
| FOX | 9.2 | 90.8 | 17 | 83 | 11.5 | 88.5 | 17.2 | 82.8 | 23.3 | 76.7 | 0.049-0.058 | 0.053 |
| ETP | 1.5 | 98.5 | 11.1 | 88.9 | 5.4 | 94.6 | 13.8 | 86.2 | 21.8 | 78.2 |  | <0.001 |
| IPM | 1.2 | 98.8 | 8 | 92 | 3.1 | 96.9 | 12.7 | 87.3 | 19.8 | 80.5 |  | <0.001 |
| AMK | 1.2 | 98.8 | 7 | 93 | 2.3 | 97.7 | 9.3 | 90.7 | 19 | 81 |  | <0.001 |
| LEV | 12 | 88 | 18 | 82 | 9.2 | 90.8 | 22 | 78 | 25.6 | 74.4 | 0.005-0.008 | 0.007 |
| SXT | 32.5 | 67.5 | 25 | 75 | 17.7 | 82.3 | 29.7 | 70.3 | 31.4 | 68.6 | 0.083-0.094 | 0.088 |
| TGC | 0 | 100 | 0 | 100 | 0 | 100 | 0 | 100 | 0 | 100 |  |  |

Abbreviation: ESBL,Extended-Spectrum Beta-Lactamases; AMC, Amoxicillin/Clavulanic acid; CSL, Cefoperazone/Sulbactam; TZP, Piperacillin/Tazobactam; CXM, Cefuroxime; CZA, Ceftazidime; CRO, Ceftriaxone; FEP, Cefepime; FOX, Cefoxitin; ETP, Ertapenem; IPM, Imipenem; AMK, Amikacin; LEV, Levofloxacin; SXT, Trimethoprim/Sulfamethoxazole; TGC, Tigecyclin.

Table 2S Antimicrobial Resistance Rates of *Pseudomonas aeruginosa* and *Acinetobacter baumannii* Isolates, 2020–2024

| **Variables** | ***Pseudomonas aeruginosa*** | | | | | | | | | | 95%CI | *p* | | |
| --- | --- | --- | --- | --- | --- | --- | --- | --- | --- | --- | --- | --- | --- | --- |
|  | 2020 (n= 24) | | 2021( n=21) | | 2022(n = 15) | | 2023(n = 17) | | 2024(n = 24) | |  |  | |  |
|  | Resistant | Non-resistant | Resistant | Non-resistant | Resistant | Non-resistant | Resistant | Non-resistant | Resistant | Non-resistant |  |  |  |  |
| CSL | 12 | 88 | 4.5 | 95.5 | 12.5 | 87.5 | 22.2 | 77.8 | 4.2 | 95.8 | 0.311-0.329 | 0.320 |  |  |
| TZP | 12 | 88 | 4.5 | 95.5 | 6.2 | 93.8 | 22.2 | 77.8 | 8.3 | 91.7 | 0.473-0.492 | 0.483 |  |  |
| CAZ | 20 | 80 | 4.5 | 95.5 | 6.2 | 93.8 | 22.2 | 77.8 | 4.2 | 95.8 | 0.157-0.171 | 0.164 |  |  |
| FEP | 4 | 96 | 4.5 | 95.5 | 0 | 100 | 11.1 | 88.9 | 4.3 | 95.7 | 0.746-0.763 | 0.755 |  |  |
| IPM | 12 | 88 | 18.2 | 81.8 | 6.2 | 93.8 | 16.7 | 83.3 | 12.5 | 87.5 | 0.859-0.872 | 0.866 |  |  |
| MEM | 8 | 92 | 22.7 | 77.3 | 0 | 100 | 16.7 | 83.3 | 13 | 87 | 0.257-0.274 | 0.265 |  |  |
| AMK | 4 | 96 | 4.5 | 95.5 | 0 | 100 | 0 | 100 | 0 | 100 | 0.882-0.895 | 0.889 |  |  |
| TOB | 5.3 | 94.7 | 4.5 | 95.5 | 0 | 100 | 5.9 | 94.1 | 0 | 100 | 0.808-0.823 | 0.815 |  |  |
| CIP | 4 | 96 | 9.1 | 90.9 | 0 | 100 | 11.1 | 88.9 | 0 | 100 | 0.276-0.293 | 0.284 |  |  |
| LVX | 4 | 96 | 9.1 | 90.9 | 6.2 | 93.8 | 22.2 | 77.8 | 0 | 100 | 0.074-0.085 | 0.079 |  |  |
| **Variables** | ***Acinetobacter baumannii*** | | | | | | | | | | 95%CI | *p* |  |  |
|  | 2020 (n=15) | | 2021(n=22) | | 2022(n=13) | | 2023(n=20) | | 2024(n=17) | |  |  |  |  |
|  | Resistant | Non-resistant | Resistant | Non-resistant | Resistant | Non-resistant | Resistant | Non-resistant | Resistant | Non-resistant |  |  |  |  |
| CSL | 20 | 80 | 22.7 | 77.3 | 46.2 | 53.8 | 70 | 30 | 52.9 | 47.4 | 0.005-0.008 | 0.006 | |  |
| TZP | 40 | 60 | 31.8 | 68.2 | 53.8 | 46.2 | 70 | 30 | 76.5 | 23.5 | 0.025-0.031 | 0.028 | |  |
| CAZ | 33.3 | 66.7 | 31.8 | 31.8 | 53.8 | 46.2 | 70 | 30 | 76.5 | 23.5 | 0.014-0.018 | 0.016 | |  |
| FEP | 33.3 | 66.7 | 22.7 | 77.3 | 53.8 | 46.2 | 70 | 30 | 64.7 | 35.3 | 0.008-0.012 | 0.010 | |  |
| IPM | 33.3 | 66.7 | 31.8 | 68.2 | 53.8 | 46.2 | 70 | 30 | 76.5 | 23.5 | 0.012-0.016 | 0.014 | |  |
| MEM | 33.3 | 66.7 | 31.8 | 68.2 | 53.8 | 46.2 | 70 | 30 | 76.5 | 23.5 | 0.012-0.016 | 0.014 | |  |
| TOB | 15.4 | 84.6 | 31.8 | 68.2 | 46.2 | 53.8 | 65 | 35 | 64.7 | 35.3 | 0.005-0.008 | 0.007 | |  |
| CIP | 33.3 | 66.7 | 31.8 | 68.2 | 53.8 | 46.2 | 70 | 30 | 76.5 | 23.5 | 0.013-0.017 | 0.015 | |  |
| LVX | 33.3 | 66.7 | 27.3 | 72.7 | 53.8 | 46.2 | 70 | 30 | 76.5 | 23.5 | 0.004-0.007 | 0.006 | |  |
| SXT | 33.3 | 66.7 | 27.3 | 72.7 | 23.1 | 76.9 | 40 | 60 | 52.9 | 47.1 | 0.422-0.441 | 0.431 | |  |
| DOX | 38.5 | 61.5 | 31.8 | 68.2 | 53.8 | 46.2 | 65 | 35 | 58.8 | 41.2 | 0.206-0.222 | 0.214 | |  |
| MNO | 23.1 | 76.9 | 13.6 | 86.4 | 15.4 | 84.6 | 20 | 80 | 0 | 100 | 0.319-0.338 | 0.328 | |  |
| TGC | 0 | 100 | 0 | 100 | 0 | 100 | 0 | 100 | 0 | 100 |  |  | |  |

Abbreviation: CSL, Cefoperazone/Sulbactam; TZP, Piperacillin/Tazobactam; CAZ, Ceftazidime; FEP, Cefepime; IPM, Imipenem; MEM, Meropenem; AMK, Amikacin; TOB, Tobramycin; CIP, Ciprofloxacin; LEV, Levofloxacin; SXT, Trimethoprim/Sulfamethoxazole; DOX, Doxycycline; MNO, Minocycline; TGC, Tigecycline.

Table 3S Antimicrobial Resistance Rates in Isolated *Staphylococci*, 2020–2024

| **Variables** | ***Staphylococcus aureus*** | | | | | | | | | | 95%CI | *p* |
| --- | --- | --- | --- | --- | --- | --- | --- | --- | --- | --- | --- | --- |
|  | 2020(n=73) | | 2021(n=71) | | 2022(n=78) | | 2023(n=62) | | 2024(n=74) | |  |  |
|  | Resistant | Non-resistant | Resistant | Non-resistant | Resistant | Non-resistant | Resistant | Non-resistant | Resistant | Non-resistant |  |  |
| PEN | 95.9 | 4.4 | 95.8 | 4.2 | 86.1 | 13.9 | 90.3 | 9.7 | 92 | 8 | 0.145-0.159 | 0.152 |
| OXA | 24.3 | 75.7 | 25 | 75 | 21.5 | 78.5 | 27.4 | 72.6 | 13.5 | 86.5 | 0.291-0.309 | 0.300 |
| GEN | 6.8 | 93.2 | 4.2 | 95.8 | 2.5 | 97.5 | 1.6 | 98.4 | 5.3 | 94.7 | 0.569-0.588 | 0.579 |
| RIF | 1.4 | 98.6 | 2.8 | 97.2 | 1.3 | 98.7 | 3.3 | 96.7 | 0 | 100 | 0.548-0.568 | 0.558 |
| LEV | 6 | 94 | 4.2 | 95.8 | 12.7 | 87.3 | 11.3 | 88.7 | 9.3 | 90.7 | 0.264-0.282 | 0.273 |
| MFX | 6 | 94 | 4.2 | 95.8 | 11.4 | 88.6 | 6.7 | 93.3 | 9.3 | 90.7 | 0.450-0.470 | 0.460 |
| SXT | 6.8 | 93.2 | 5.6 | 94.4 | 3.8 | 96.2 | 6.5 | 93.6 | 10.7 | 89.3 | 0.571-0.590 | 0.580 |
| CLI | 21.6 | 78.4 | 19.4 | 80.6 | 30.4 | 69.6 | 25.8 | 74.2 | 28 | 72 | 0.573-0.593 | 0.583 |
| ERY | 35.1 | 64.9 | 26.4 | 73.6 | 32.9 | 67.1 | 25.8 | 74.2 | 29.3 | 70.7 | 0.665-0.684 | 0.675 |
| LNZ | 0 | 100 | 0 | 100 | 0 | 100 | 0 | 100 | 0 | 100 |  |  |
| VAN | 0 | 100 | 0 | 100 | 0 | 100 | 0 | 100 | 0 | 100 |  |  |
| TGC | 0 | 100 | 0 | 100 | 0 | 100 | 0 | 100 | 0 | 100 |  |  |
| **Variables** | ***Coagulase-negative staphylococci*** | | | | | | | | | | 95%CI | *p* |
|  | 2020(n=87) | | 2021(n=84) | | 2022(n=104) | | 2023(n=115) | | 2024(n=145) | |  |  |
|  | Resistant | Non-resistant | Resistant | Non-resistant | Resistant | Non-resistant | Resistant | Non-resistant | Resistant | Non-resistant |  |  |
| PEN | 96.6 | 3.4 | 94 | 6 | 95.2 | 4.8 | 95.7 | 4.3 | 93.2 | 6.8 | 0.819-0.834 | 0.827 |
| OXA | 85.1 | 14.9 | 81.9 | 18.1 | 76 | 24 | 76.7 | 23.3 | 74.7 | 25.3 | 0.297-0.315 | 0.306 |
| GEN | 23 | 77 | 19 | 81 | 10.5 | 89.5 | 16.4 | 83.6 | 11 | 89 | 0.060-0.070 | 0.065 |
| RIF | 16.1 | 83.9 | 14.5 | 85.5 | 11.5 | 88.5 | 12.4 | 87.6 | 9.7 | 90.3 | 0.645-0.663 | 0.654 |
| LEV | 59.3 | 40.7 | 51.2 | 48.8 | 50.5 | 49.5 | 62.9 | 37.4 | 54.8 | 45.2 | 0.336-0.355 | 0.346 |
| MFX | 44.1 | 55.9 | 31.3 | 68.7 | 38.5 | 61.8 | 48.7 | 51.3 | 38.6 | 61.4 | 0.121-0.135 | 0.128 |
| SXT | 39.1 | 60.9 | 28.6 | 71.4 | 20 | 80 | 19 | 81 | 14.4 | 85.6 |  | <0.001 |
| CLI | 44.8 | 55.2 | 38.1 | 61.9 | 45.7 | 54.3 | 46.6 | 53.4 | 49.3 | 50.7 | 0.032-0.039 | 0.035 |
| ERY | 71.3 | 28.7 | 65.5 | 34.5 | 63.8 | 36.2 | 60.3 | 39.7 | 68.5 | 31.5 | 0.466-0.486 | 0.476 |
| LNZ | 0 | 100 | 0 | 100 | 1 | 99 | 1.7 | 98.3 | 1.4 | 98.6 | 0.759-0.776 | 0.768 |
| VAN | 0 | 100 | 0 | 100 | 0 | 100 | 0 | 100 | 0 | 100 |  |  |
| TGC | 0 | 100 | 0 | 100 | 0 | 100 | 0 | 100 | 0 | 100 |  |  |

Abbreviation: PEN, Penicillin; OXA, Oxacillin; GEN, Gentamicin; RIF, Rifampicin; LVX, Levofloxacin; MFX, Moxifloxacin; SXT, Trimethoprim/Sulfamethoxazole; CLI, Clindamycin; ERY, Erythromycin; LNZ, Linezolid; VAN, Vancomycin; TGC, Tigecycline.

Table 4S Antimicrobial Resistance Rates Among Isolated *Enterococci*, 2020–2024

| **Variables** | ***Enterococcus faecium*** | | | | | | | | | | **95%CI** | ***p*** |
| --- | --- | --- | --- | --- | --- | --- | --- | --- | --- | --- | --- | --- |
|  | 2020(n=10) | | 2021(n=16) | | 2022(n=13) | | 2023(n=28) | | 2024(n=21) | |  |  |
|  | Resistant | Non-resistant | Resistant | Non-resistant | Resistant | Non-resistant | Resistant | Non-resistant | Resistant | Non-resistant |  |  |
| PEN | 62.5 | 37.5 | 93.8 | 6.2 | 84.6 | 15.4 | 96.4 | 3.6 | 90.5 | 9.5 | 0.027-0.034 | 0.030 |
| AMP | 70 | 30 | 93.8 | 6.2 | 76.9 | 23.1 | 96.4 | 3.6 | 90.5 | 9.5 | 0.095-0.107 | 0.101 |
| GEH | 10 | 90 | 25 | 75 | 23.1 | 76.9 | 40.7 | 59.3 | 66.7 | 33.3 | 0.009-0.013 | 0.011 |
| LVX | 62.5 | 37.5 | 93.8 | 6.2 | 84.6 | 15.4 | 96.4 | 3.6 | 90.5 | 9.5 | 0.039-0.047 | 0.043 |
| ERY | 90 | 10 | 87.5 | 12.5 | 84.6 | 15.4 | 85.2 | 14.8 | 71.4 | 28.6 | 0.667-0.685 | 0.676 |
| LNZ | 10 | 90 | 6.2 | 93.8 | 0 | 100 | 3.6 | 96.4 | 4.8 | 95.2 | 0.836-0.850 | 0.843 |
| VAN | 0 | 100 | 0 | 100 | 7.7 | 92.3 | 7.1 | 92.9 | 9.5 | 90.5 | 0.790-0.805 | 0.798 |
| **Variables** | ***Enterococcus faecalis*** | | | | | | | | | | **95%CI** | ***p*** |
|  | 2020(n=12) | | 2021(n=16) | | 2022(n=15) | | 2023(n=11) | | 2024(n=7) | |  |  |
|  | Resistant | Non-resistant | Resistant | Non-resistant | Resistant | Non-resistant | Resistant | Non-resistant | Resistant | Non-resistant |  |  |
| PEN | 0 | 100 | 12.5 | 87.5 | 0 | 100 | 9.1 | 90.9 | 0 | 100 | 0.494-0.514 | 0.504 |
| AMP | 0 | 100 | 0 | 100 | 0 | 100 | 9.1 | 90.9 | 0 | 100 | 0.494-0.514 | 0.504 |
| GEH | 0 | 100 | 12.5 | 87.5 | 20 | 80 | 27.3 | 72.7 | 57.1 | 62.9 | 0.030-0.037 | 0.033 |
| LVX | 0 | 100 | 31.2 | 68.8 | 6.7 | 93.3 | 36.4 | 83.6 | 14.3 | 85.7 | 0.058-0.067 | 0.063 |
| ERY | 46.2 | 53.8 | 62.5 | 37.5 | 40 | 60 | 45.5 | 54.5 | 71.4 | 50.6 | 0.591-0.61 | 0.601 |
| LNZ | 0 | 100 | 6.2 | 93.8 | 6.7 | 93.3 | 9.1 | 90.9 | 28.6 | 71.4 | 0.297-0.315 | 0.306 |
| VAN | 0 | 100 | 0 | 100 | 0 | 100 | 0 | 100 | 0 | 100 |  |  |

Notes: PEN, Penicillin; AMP, Ampicillin; GEH, high-level gentamicin resistance; LEV, Levofloxacin; ERY, Erythromycin; LNZ, Linezolid, VAN, Vancomycin.
